# The Effectiveness of Web-Based Asthma Self-Management System, My Asthma Portal (MAP): A Pilot Randomized Controlled Trial

**DOI:** 10.2196/jmir.5866

**Published:** 2016-12-01

**Authors:** Sara Ahmed, Pierre Ernst, Susan J Bartlett, Marie-France Valois, Tasneem Zaihra, Guy Paré, Roland Grad, Owis Eilayyan, Robert Perreault, Robyn Tamblyn

**Affiliations:** ^1^Division of Clinical EpidemiologyMcGill University Health CenterMcGill UniversityMontreal, QCCanada; ^2^School of Physical & Occupational TherapyFaculty of MedicineMcGill UniversityMontreal, QCCanada; ^3^Department of MedicineFaculty of MedicineMcGill UniversityMontreal, QCCanada; ^4^Centre de recherche interdisciplinaire en réadaptation (CRIR), Constance Lethbridge Rehabilitation CenterMontreal, QCCanada; ^5^Centre for Clinical EpidemiologyLady Davis Research InstituteJewish General HospitalMcGill UniversityMontreal, QCCanada; ^6^The College at BrockportDepartment of MathematicsState University of New YorkBrockport,, NYUnited States; ^7^Department of Information TechnologyHEC MontrealMontreal, QCCanada; ^8^Department of Family MedicineFaculty of MedicineMcGill UniversityMontreal, QCCanada; ^9^Department of PsychiatryFaculty of MedicineMcGill UniversityMontreal, QCCanada; ^10^Biostatistics and Occupational Health and the Clinical and Health Informatics Research GroupDepartment of EpidemiologyMcGill UniversityMontreal, QCCanada

**Keywords:** Internet, nursing, case management, self-care, asthma, quality of life

## Abstract

**Background:**

Whether Web-based technologies can improve disease self-management is uncertain. My Asthma Portal (MAP) is a Web-based self-management support system that couples evidence-based behavioral change components (self-monitoring of symptoms, physical activity, and medication adherence) with real-time monitoring, feedback, and support from a nurse case manager.

**Objective:**

The aim of this study was to compare the impact of access to a Web-based asthma self-management patient portal linked to a case-management system (MAP) over 6 months compared with usual care on asthma control and quality of life.

**Methods:**

A multicenter, parallel, 2-arm, pilot, randomized controlled trial was conducted with 100 adults with confirmed diagnosis of asthma from 2 specialty clinics. Asthma control was measured using an algorithm based on overuse of fast-acting bronchodilators and emergency department visits, and asthma-related quality of life was assessed using the Mini-Asthma Quality of Life Questionnaire (MAQLQ). Secondary mediating outcomes included asthma symptoms, depressive symptoms, self-efficacy, and beliefs about medication. Process evaluations were also included.

**Results:**

A total of 49 individuals were randomized to MAP and 51 to usual care. Compared with usual care, participants in the intervention group reported significantly higher asthma quality of life (mean change 0.61, 95% CI 0.03 to 1.19), and the change in asthma quality of life for the intervention group between baseline and 3 months (mean change 0.66, 95% CI 0.35 to 0.98) was not seen in the control group. No significant differences in asthma quality of life were found between the intervention and control groups at 6 (mean change 0.46, 95% CI –0.12 to 1.05) and 9 months (mean change 0.39, 95% CI –0.2 to 0.98). For poor control status, there was no significant effect of group, time, or group by time. For all self-reported measures, the intervention group had a significantly higher proportion of individuals, demonstrating a minimal clinically meaningful improvement compared with the usual care group.

**Conclusions:**

This study supported the use of MAP to enhance asthma quality of life but not asthma control as measured by an administrative database. Implementation of MAP beyond 6 months with tailored protocols for monitoring symptoms and health behaviors as individuals’ knowledge and self-management skills improve may result in long-term gains in asthma control.

**ClinicalTrial:**

International Standard Randomized Controlled Trial Number (ISRCTN): 34326236; http://www.isrctn.com/ISRCTN34326236 (Archived by Webcite at http://www.webcitation.org/6mGxoI1R7).

## Introduction

The episodic nature of asthma symptoms and exacerbations makes effective self-management of the condition an imperative. Ongoing self-monitoring of asthma control coupled with an individualized written asthma action plan is required to allow patients to quickly identify and address poor disease control and mild exacerbations by increasing medication or contacting a physician [1]. However, less than 40% of individuals with asthma regularly monitor their symptoms [2] and even fewer initiate their prescribed action plan at the first signs of an exacerbation [3,4]. Further, ongoing self-monitoring and self-management is in part related to the extent to which patients’ treating physician and care team provide review and feedback [5-7]. In addition, many patients have difficulty recognizing signs of early deterioration and often fail to utilize their asthma action plans and access their physicians in a timely manner to control worsening asthma status [8,9].

Providing ongoing communication and self-management support is challenging given limited clinical resources and time [10,11]. Health information technology may offer unique opportunities to facilitate integrated self-management support by providing a means for ongoing monitoring and feedback combined with 2-way communication and information exchange between the patient and his or her care team between visits. Providing patients with chronic conditions timely access to health information and personalized alerts, when there is a need for action, may empower individuals to self-manage more effectively by facilitating and reinforcing health behavior change.

A review that investigated the effectiveness of using computers to deliver patient self-management programs for chronic illness found that there was insufficient evidence to determine whether computer-enabled interventions were effective compared with no intervention or usual care [10]. Although the results have been inconsistent, a systematic review of computer-based interventions for asthma supported that these interventions may be effective in improving knowledge, reducing activity limitations, improving markers of self-management, improving quality of life, and optimizing medication use [12]. However, 21% of trials were among children. Most studies failed to report information on the socioeconomic status of participants, and few included individuals over the age of 50 years, limiting generalizability. Furthermore, most trials have only evaluated isolated components required for self-management of asthma, restricting the comprehensiveness of the self-management support received by the patients, and only one study had the support of a case manager to intervene when needed. Finally, whereas patient report is necessary to evaluate the impact of behavioral interventions, previous studies in asthma have not included an evaluation of asthma control independent of participant reporting such as the combined need for emergency care and overuse of rescue medications [13,14]. These outcomes provide necessary information to assess the impact of Web-based interventions on longer-term outcomes, less influenced by daily fluctuations in symptoms, and address the impact of disease on individuals’ consumption of health care services and related costs.

More evidence is needed to determine whether Web-based self-management support systems are helpful in clinical practice and can lead to better outcomes. In addition, more information is needed about the proportion of patients who are willing and able to use a Web-based self-management tool and the functionalities that are most frequently accessed. The primary objective of this study was to compare the impact of access to a customized personal Web-based self-management asthma patient portal, My Asthma Portal (MAP), linked to a case-management system over 6 months compared with usual care on asthma control and asthma quality of life. We hypothesized that at 6 months, use of MAP would be associated with improved asthma control and asthma quality of life.

## Methods

### Ethical Considerations

The McGill University Health Center ethics committee provided approval for this study. Research nurses provided patients with an information brochure, and patients were invited to speak to the research assistant for further information. The research assistant obtained informed patient consent. The detailed protocol is available elsewhere [15].

### Setting and Participants

Participants were recruited from pulmonary clinics in 2 tertiary care hospitals located in Montreal, Canada. Participants were eligible if they were aged between 18 and 69 years, had a physician diagnosis of asthma, were prescribed at least one rescue medication, classified as having poor asthma control at the time of recruitment by their treating physician, had access to the Internet, reported smoking <20 pack-years, and were fluent in English or French. We excluded individuals with a diagnosis of chronic obstructive pulmonary disease or other serious medical diagnoses (eg, lung cancer), and those with severe mobility limitations. Potentially eligible participants were identified and approached by their pulmonologist or the nursing staff. The research assistant provided additional information about the study, responded to questions, and obtained written consent.

### Study Design

The study was a 6-month, multicenter, parallel, 2-arm, pilot, randomized controlled trial (ISRCTN34326236). Participants were randomly assigned to 1 of the 2 conditions: (1) MAP access or (2) usual care. Randomization occurred at the individual patient level in 2 participating pulmonary clinics. Treatment allocation was done by random permutation within blocks with block sizes of 4 and 6 using a computerized algorithm.

### Interventions

#### Intervention: My Asthma Portal

MAP was developed to support patient self-management and facilitate communication with the care team between visits using an iterative design process based on behavior change and self-efficacy theory [15]. The impact of self-management on health outcomes is thought to occur primarily through changes in health behaviors by helping patients develop the confidence to engage in tasks and acquire core knowledge and skills aimed at helping them better manage their health. Therefore, the features within MAP are based on increasing individuals’ knowledge as well as their confidence to carry out the behaviors that are necessary for patients to improve their asthma health. These features are summarized in Table 1 of the protocol publication [15].

Participants were given a MAP username and password, advised that they could access the site from anywhere (eg, home, work, library), and asked to login at least once per week for the 6 months of the study (access to MAP ended after this period). The log-in frequency was decided based on clinician feedback, the fact that symptoms are monitored over 1-week period, and patient feedback on what log-in frequency they believed was feasible as obtained during the development phase. Furthermore, based on a systematic review, the typical Web-based intervention is used once a week [16]. During the first log-in, patients provided basic demographic and health information (eg, smoking status, allergies, and triggers) and selected their own initial health goals for action.

Within MAP, participants could (1) view their personal health information (eg, asthma medications, other health problems); (2) view general asthma information through links to specific educational websites (Learning Center) and receive information tailored to identified knowledge gaps (eg, current medications); and (3) monitor and receive feedback regarding current self-management practices.

Initial feedback was generated automatically from the MAP system using monitoring information entered by the participant and data from the provincial administrative database sent to MAP from the Medical Office for the twenty first century (MOXXI), an electronic health record with a prescription and computerized drug and disease management system. A summary of the logic for color-coding and recommendations offered has been previously summarized [17] and is presented in Multimedia Appendix 1. The color-coding was based on the Canadian asthma guidelines for symptom management [1]. An email alert was sent to patients under the following conditions: (1) when suboptimal asthma control was identified by the system and the participant indicated they did not initiate activities outlined in their action plan; (2) if the action plan was updated by the nurse as prescribed by the patient’s physician; and (3) if they had not logged in for at least seven days. The participant was given an opportunity to act on these alerts; if no action had occurred within 48 hours, the alert status was escalated to notify the nurse case manager.

Participants communicated and received support as needed from a nurse case manager using MAP. The nurse case-management system was designed to (1) quickly identify patients that may require immediate care; (2) collate relevant medical and monitoring information for each participant; and (3) document case-management information, including interactions (phone and emails), along with advice and interventions provided by the case manager. Upon receiving this notice, the nurse followed up with the participant within 24 hours through the MAP mail system or by telephone. Additional alerts were sent to the nurse case manager when a new participant was enrolled and needed their prescribed action plan reviewed, the participant indicated lack of understanding of the action plan, or there was a change in medication use based on the self-reported adherence monitoring assessment.

#### Control Intervention: Usual Care

The control group did not have access to MAP. All participants in the control and intervention groups continued to receive ongoing asthma care from their pulmonologist throughout the trial, and an asthma nurse provided education and follow-up sessions as needed. Topics (similar to those found in the static learning module of MAP) included the importance of avoiding triggers, taking all asthma medications as prescribed, and using the written action plan. The asthma nurse conducted follow-up phone calls between visits, when appropriate (ie, missed appointments, to clarify aspects of the action plan or prescribed asthma medications).

### Measures

#### Process Evaluation

Automated audit trails (computer logs) were used to evaluate MAP usage rates. Use was defined as the frequency and intensity, mainly the number of times and minutes patients spent logged into the system. Patterns of usage included review of the days/week and amount of time that patients used the system. Features used included the number of times and topic of the messages sent to the nurse case manager.

We also evaluated acceptability and attitude of participants toward the Web portal. We used adapted versions of instruments associated with the technology acceptance model (TAM) to assess perceptions of usefulness and ease of use [18-21]. Items were scored from 1 (strongly disagree) to 5 (strongly agree). In other studies, reliability of ratings ranged from 0.92 to 0.98 for usefulness and 0.88 to 0.94 for ease of use [18,20]. Perceived usefulness and ease of use have also been found to be significantly correlated with both self-reported current usage (*r*=.63, *r*=.45, respectively) and self-predicted future usage (*r*=.85, *r*=.59, respectively) [18].

#### Primary Outcomes

##### Asthma Control at 6 Months

Asthma control at 6 months was evaluated by examining potential overuse (yes/no) of rescue fast-acting bronchodilators (FABA; ie, beta 2-agonists) based on units dispensed for prescriptions as recorded in the provincial drug database that covers drugs dispensed for individuals who are provincially insured, medical services provided, emergency department (ED) visits, and hospitalizations. Excessive use of FABA was chosen because it is associated with an increased risk of hospitalization and asthma mortality [22]. Based on a previously validated algorithm, asthma was classified as poorly controlled if the sum of the quantity for all FABAs dispensed was >500 doses of salbutamol 100 mcg, 2 inhalations at a time, or the equivalent for other FABAs in the last 6 months of follow-up [1], or the participants visited an ED for a pulmonary-related problem in the last 6 months. To evaluate asthma control at 3 months (halfway), half the amount of FABA was used to calculate overuse and ED visits were evaluated over the 3-month period.

##### Asthma Quality of Life

The Mini-Asthma Quality of Life Questionnaire (MAQLQ) evaluates symptoms, emotions, exposure to environmental stimuli, and activity limitations [17,23] using a 7-point Likert scale. The MAQLQ was chosen because it is internally consistent (interclass correlation coefficient=0.83), correlates highly with the 32-item version (*r*=.90) and moderately (*r*=.69) with the Asthma Control Questionnaire [23,24], and is sensitive to change (*P*<.001) [23].

#### Secondary Outcomes and Baseline Variables

##### Demographic Information

Demographic information such as sex, age, education, and socioeconomic status based on postal code were extracted from the baseline questionnaires. Participants reported on education level at baseline.

##### The Chronic Disease Self-Efficacy Scale

The Chronic Disease Self-Efficacy Scale [25] has been shown to have adequate psychometric properties in patients with chronic conditions, with internal consistency ranging from .77 to .92 and test-retest reliability .72 to .89 [25,26], and was adapted in this study to assess asthma self-efficacy. Participants rated their level of confidence (1=not confident at all to 10=very confident) with respect to taking medications as prescribed, recognizing and acting on symptoms consistent with deteriorating asthma status, knowing when to initiate their action plan, eating a healthy diet, and participating regularly in physical activity.

##### The Asthma Control Test

The Asthma Control Test (ACT), a 5-point patient-administered survey for assessing asthma control, evaluates patient perceptions of asthma control [27,28]. Patients rate asthma symptoms over the past 4 weeks as well as their overall level of asthma control. The ACT has been found to be internally consistent (Cronbach alpha=.85) [28] and to have moderate test-retest reliability (intracluster correlation=0.77).

##### Beliefs About Medicines Questionnaire

Patient’s beliefs about their medicines were evaluated using the Beliefs about Medicines Questionnaire (BMQ) [17,29]. The BMQ assesses beliefs about the “necessity” of prescribed medication for controlling their illness as well as “concerns” about the potential adverse consequences. The necessity-concerns differential score is calculated by subtracting the concerns score from the necessity score (range –20 to 20) [30]. A positive differential score indicates that the patient has stronger beliefs in the necessity of medications compared with concerns and vice versa in the case of a negative score. The 2 BMQ subscales are internally consistent (Cronbach alpha for necessity and concern: 0.87 and 0.78, respectively) [30].

##### Patient Health Questionnaire

Patient Health Questionnaire (PHQ-9) is a tool based on the Diagnostic and Statistical Manual of Mental Disorders, 4th Edition, criteria for identifying depressive symptoms that has been validated and widely used in a number of patient populations, including older adults and patients with asthma [31,32]. A PHQ-9 score of 10 has an 88% sensitivity and 88% specificity for symptoms of major depression. Asthmatics with a PHQ-9 score of greater than or equal to 10 were classified as having symptoms suggestive of moderate or more severe depression.

##### EuroQol Visual Analogue Scale

Health status was evaluated with the EuroQol visual analogue scale (EQ-VAS). Patients were asked to rate their current health state on a 0 (worst imaginable health state) to 100 (best imaginable health state) scale [33].

##### Asthma-Related ED Visits or Hospitalizations

Asthma-related ED visits or hospitalizations were extracted from the Quebec provincial insurance database using validated algorithms [34]. Information on the date, type, provider, and location (eg, inpatient, emergency, clinic) for all fee-for-service medical services (~86% of all services) [35] is available for all Quebec residents.

All outcomes were collected at 3 months (halfway) and 6 months (end) with follow-up 9 months later to evaluate persistent effects. Questionnaires were administered in the clinic at baseline and via mail thereafter.

### Statistical Analyses

The study was designed to have 80% power (alpha=.05) to detect a 10% difference in proportions of patients whose asthma was classified as out of control. This required a total sample size of 67 participants. To account for attrition and loss to follow-up, an additional 33 subjects were recruited (total 100; 50 per arm). This sample size also allowed us to detect a clinically meaningful difference of 0.5 (SD 0.61) on the MAQLQ scores [17].

Descriptive statistics, including means for continuous variables and proportions for categorical variables, were used to summarize participant characteristics. Analyses were conducted using an intention-to-treat analysis.

A generalized estimating equation model [36] was used to compare the proportions identified as having poor asthma control between groups. The clustering effect was at the level of the patient to account for multiple observations on the same person over time. Mixed-effects linear regression [36] modeling was used to estimate the difference in asthma quality of life between groups. For both analyses, patient was the unit of analysis. An auto-regressive by a degree of 1 (AR(1)) correlation structure was used for the asthma control outcome, and compound symmetry (CS) correlation structure was used for the asthma quality of life outcome to account for multiple observations for the same patient [36]. Both types of analyses were performed testing the effects of time, intervention, and an interaction between time and intervention (as fixed effects in the case of mixed-effects models). Analyses were conducted with SAS version 9.3 (SAS Institute Inc) using PROC GENMOD (binary outcome), and PROC MIXED (continuous outcomes). Models were adjusted for number of asthma medications, sex, age, education level, and self-reported health. Sensitivity analyses were conducted to evaluate the effects of missing data by imputing missing data using the Markov chain Monte Carlo method [37] with relative efficiency of 98% in SAS (PROC MI, PROC MIANALYZE).

Secondary analyses were conducted to evaluate the mediating effects of depressive symptoms (PHQ), self-efficacy, beliefs about asthma medication (BMQ), and asthma control (ACT). In addition, the MAQLQ was added to the model with poorly controlled asthma as the outcome and asthma control status for the model with MAQLQ as the outcome.

Furthermore, the proportion of individuals in each group who achieved a minimal clinically important difference (MCID) between follow-up evaluations on self-report measures was also examined. The estimated MCID of the MAQLQ was 0.5 [2,3], the EQ-VAS was 5, and the PHQ-9 was 5 [38]. As this has not been established for the ACT and self-efficacy scales, we used the convention of 0.5 SD to define a MCID [39].

## Results

### Participants

**Figure 1:**
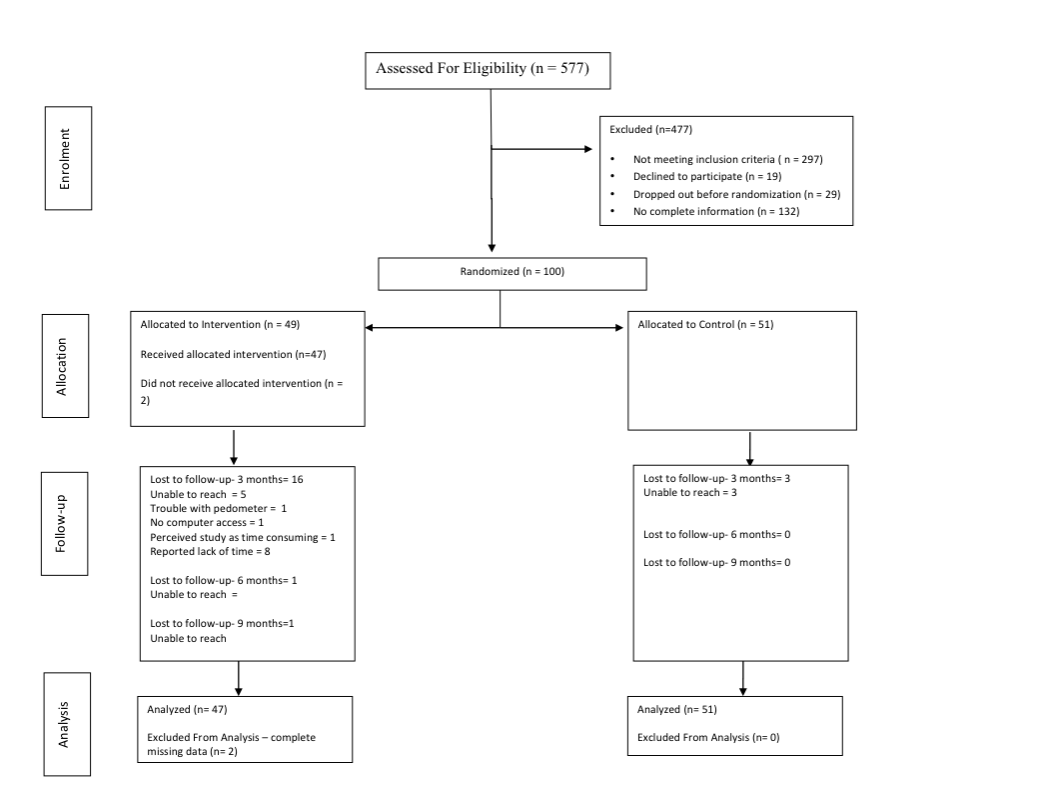
Consort diagram of participants in the My Asthma Portal (MAP) trial.

Of the total patients screened, 17.3% (100/577) met eligibility criteria, consented to participate, and were randomized. The remaining 83% did not meet the inclusion criteria (62.3%, 297/477), declined to participate (4.0%, 19/477), dropped out before randomization (6.1%, 29/477), or did not have sufficient information to approach for recruitment (27.7%, 132/477; Figure 1). A total of 51 individuals were randomized to the control group and 49 to the intervention group. Table 1 presents the baseline characteristics for both the groups.

The distribution of characteristics between groups was similar; however, the proportion of individuals with a university degree and in the 0-39 age group was higher in the intervention group as compared with the control group (Table 1). Whereas the proportion of individuals who had one or more ED visits or hospitalizations for a pulmonary problem in 3 months before enrolment was similar between groups, in the 3 months before randomization, participants in usual care were more likely to overuse FABA in the 9 months before enrolment compared with those in the intervention group (12% vs 4%, respectively), although this difference was not statistically significant.

**Table 1:** Baseline characteristics of participants in the intervention and control groups.

Characteristic		Intervention (n=47)	Control (n=51)	*P* value
		n (%)	n (%)	
**Sex**				
	Female	32 (68)	33 (65)	.72
**Age group (years)**				
	0-39	20 (43)	14 (27)	.25
	40-49	10 (21)	13 (25)	
	50-59	12 (26)	15 (29)	
	>60	5 (11)	9 (18)	
**Smoking status**				
	Never smoker	26 (55)	26 (51)	.91
	Current smoker	5 (11)	7 (14)	
	Ex-smoker	15 (32)	16 (31)	
	Missing	1 (2)	2 (4)	
**Education**				
	>Higher school	32 (68)	26 (51)	.38
	High school equivalent	13 (28)	22 (43)	
	<High school	1 (2)	1 (2)	
	Missing	1 (2)	2 (4)	
**Self-reported health**				
	Excellent or very good	8 (17)	14 (27)	.31
	Good	24 (51)	19 (37)	
	Fair or poor	15 (32)	18 (35)	
**Depression-PHQ^b^**				
	None (0-4)	22 (47)	27 (53)	.52
	Mild (5-9)	19 (40)	13 (25)	
	Moderate (10-14)	2 (4)	5 (10)	
	Moderately severe or severe (15-19 or 20-7)	4 (8)	6 (12)	
**Prescribed action plan for asthma at baseline**				
	Yes	33 (70)	41 (80)	.35
	No	13 (28)	10 (20)	
	Missing	1 (2)	0 (0)	
**Number of asthma medications**				
	1	7 (15)	14 (27)	.25
	2	19 (40)	17 (33)	
	3	12 (26)	6 (12)	
	≥4	6 (13)	10 (20)	
	Missing	3 (6)	4 (8)	
**Poorly controlled asthma**				
	No	38 (81)	36 (71)	.34
	Yes	7 (15)	9 (18)	
	Missing	2 (4)	6 (12)	
**ED^c^ visits due to a respiratory problem**				
	0	37 (79)	38 (75)	.32
	1	6 (13)	3 (6)	
	≥2	2 (4)	4 (8)	
	Missing	2 (4)	6 (12)	
**FABA overuse^a^**				
	0	43 (91)	39 (76)	.48
	1	2 (4)	6 (12)	
	Missing	2 (4)	6 (12)	
**Number of hospitalizations due to respiratory issues**				
	0	44 (94)	43 (84)	.42
	1	1 (2)	1 (2)	
	≥2	0 (0)	1 (2)	
	Missing	2 (4)	6 (12)	

^a^PHQ: Patient Health Questionnaire.

^b^FABA (fast-acting bronchodilators) overuse: if the sum of the quantity for all FABAs dispensed was >750+ mg doses of salbutamol 100 mcg, 2 inhalations at a time, or the equivalent for other FABAs over a 9-month period.

^c^ED: emergency department.

Of the 100 individuals who were randomized, 16 were lost to follow-up at 3 months in the intervention group. Five individuals could no longer be reached, 1 reported having trouble with the pedometer and did not log in, 1 no longer had access to a computer, 1 reported that the study was time-consuming, and the remaining 8 individuals reported lack of time. In addition, one individual at 6 months and another at 9 months postrandomization could no longer be reached. Among these individuals, 5 did not log in to MAP at all, and the remainder logged in 1 to 36 times. Three individuals in the control group were lost to follow-up at 3 months postrandomization.

### Process Evaluation

**Figure 2:**
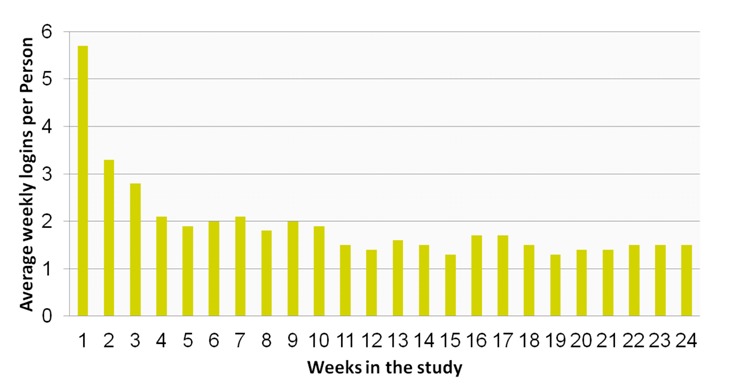
Average number of logins by week over the 6-month study period.

**Figure 3:**
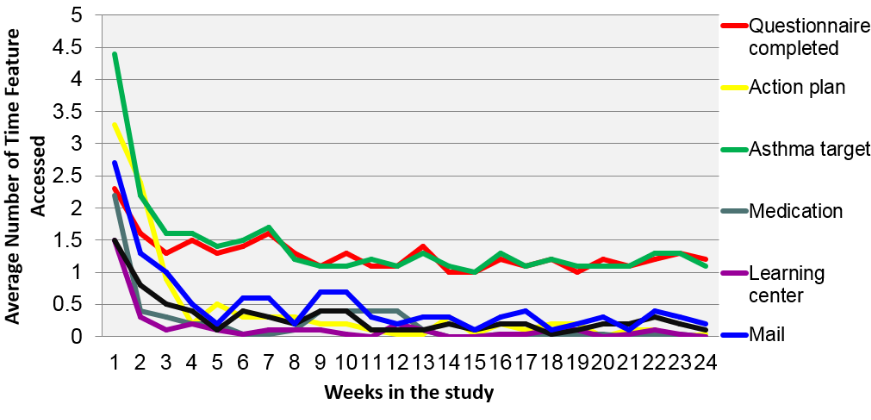
Usage of My Asthma Portal (MAP) features over time.

Access to MAP during the first 3 months ranged from 1 to 69 (mean 24, SD 16), and 0 to 38 between 3 and 6 months; among the 47 individuals who received the intervention, 16% had no logins between 3 and 6 months (Figure 2). Logins were more frequent in the first 4 weeks (mean 12, SD 8) and then tapered off thereafter (mean 36, SD 42). The average time spent logged into MAP was 7 minutes/login (range 20 seconds to 2 hours). The most frequently accessed components of the portal were the monitoring questionnaire and the asthma feedback center, with an average of 4.5 views in the first week, 2.2 in the second week, 1.5 from week 2 to week 7, and then 1 time per week for the remainder of the trial (Figure 3). The least frequently accessed component was the static Learning Center. In all instances when a monitoring questionnaire was started, it was completed. Among those who logged in, the proportion of participants who logged in at least once per week was the lowest in week 19 (56%) and the highest in week 1 (100%). Four individuals did not log in at all during the 6-month study period. Their characteristics were similar to those who used MAP except for depressive symptoms, which on average were lower (mean 5, SD 3) in those who did not log in compared with those who used MAP (mean 6, SD 5).

The most frequent reasons for initiating an email by the patient or the nurse was to discuss medications (30%), clarify the action plan (18%), monitor MAP feedback (15%), and conduct follow-ups related to control status (5%). About 61% of alerts to the nurse were related to medication adherence, 17% indicated a need to review the action plan, 11% were because the patient did not log in, and 7% were related to delayed initiation of the action plan by patients.

Usefulness ratings on the TAM were essentially unchanged at baseline and 6 months. A similar pattern was found for the response to the item “I intend to use My Asthma Web portal weekly to help manage my Asthma.”

### Impact of MAP on Asthma Control Using Administrative Data and Health-Related Quality of Life

Multimedia Appendix 2 presents the scores for asthma control status and health-related quality of life (MAQLQ) over time by group. After adjustment for sex, age, asthma medications, education, and self-reported health, a significant improvement in MAQLQ score was evident between baseline and 3 months for the intervention group (mean change 0.67, 95% CI 0.36 to 0.98). Differences were not statistically significant for the intervention group between 3 and 6 months (mean change –0.01, 95% CI –0.35 to 0.32) or 6 and 9 months (mean change –0.12, 95% CI –0.46 to 0.22). There were no statistically significant differences over time for the control group. Further, there were no significant differences between groups over time for both MAQLQ and poor control status.

When models were reanalyzed with imputed data, the difference in MAQLQ scores between baseline and 3 months for the intervention group was still statistically significant after adjustment for sex, age, asthma medications, education, and self-reported health (mean change 0.52, 95% CI 0.20 to 0.84). In addition, a trend of decreasing scores between the 6 and 9 months was found (mean change –0.35, 95% CI –0.66 to –0.04) for the intervention group. No other statistically significant differences were found between time periods or between groups for MAQLQ scores. There was no significant effect of group, time, or group by time adjusted for sex, age, asthma medications, education, and self-reported health found for poor control status.

The results for differences in MAQLQ scores over time and between groups were similar with adjustment for depression (PHQ), self-efficacy, beliefs about asthma medication (BMQ), self-reported asthma control (ACT), and poor control status at baseline. In addition, depression (PHQ) (mean change –0.27, 95% CI –0.37 to –0.18 for a change of 5 units), self-efficacy (mean change 0.24, 95% CI 0.16 to 0.32), and self-reported asthma control symptoms (ACT) (mean change –0.25, 95% CI –0.30 to –0.20) were all significantly associated with asthma quality of life (MAQLQ). No significant effect of group, time, or group by time or for the explanatory variables was found for poor control status.

**Figure 4:**
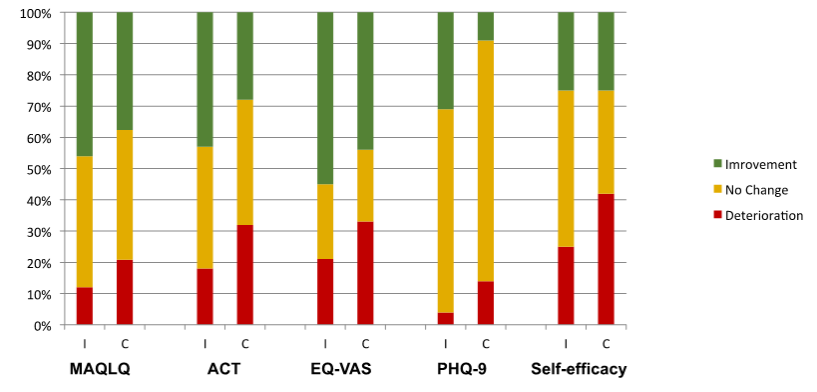
Minimal clinically important difference at 6 months on the patient reported outcomes. MAQLQ: Mini-Asthma Quality of Life Questionnaire; ACT: Asthma Control Test; EQ-VAS: EuroQol visual analogue scale; PHQ: Patient Health Questionnaire.

Figure 4 shows the proportion of individuals who showed a clinically meaningful improvement, no improvement, or deterioration on the patient-reported outcomes over the 6-month intervention period by group. For all measures, the intervention group had a higher proportion of individuals demonstrating a minimally clinically important difference compared with the control group. However, only the difference in depression (PHQ) at 6 months was statistically significant.

## Discussion

### Principal Findings

Our study found significant improvements in asthma quality of life over the initial 3 months among individuals using a Web-based asthma self-management intervention compared with those in the control group. There was also a higher proportion of individuals who had an improvement in depression, self-efficacy, and self-reported asthma control over time in the intervention group compared with the control group, suggesting that these were important mediating effects of the MAP intervention on improving asthma quality of life. However, there was no significant effect of the intervention on asthma control over time or for differences between groups in asthma control and asthma-related quality of life.

The proportion of poor control status seemed to worsen slightly in the intervention group and to improve in the usual care group during the study. This was not surprising, as the control group might also benefit from being included in a study. This suggested, however, that when patients reduced their interaction with MAP it might not be because they had learned how to self-manage their condition. Other potential reasons might be a loss of interest in the system, or feeling overwhelmed and unable to log in when symptoms worsened. Identifying mechanisms to keep individuals engaged and motivated to use systems such as MAP, which are relatively simple to integrate into daily activities, is important. Strategies found to be effective to increase the use of Web-based systems include primary task support that includes delivering the intervention in a stepwise approach as individuals’ knowledge and behavioral changes progress and matching content and features of the intervention to the individuals’ needs and health [16].

Ongoing access by participants using MAP supports the feasibility of using the system to deliver self-management for individuals who accept to start using the system. Also, the fact that logins decreased over time may reflect that once individuals learned about their medications and action plan they found less benefits of logging in to report symptoms and receive feedback that they became more familiar with over time. This reflects the importance of tailoring content and features of Web-based interventions over time to changes in individuals’ knowledge, confidence, and symptoms as supported by previous reviews of self-management technology-based interventions [12,40].

Similar to our findings, a recent review of systematic reviews [12,40] showed that the 2 of the 7 studies that were conducted in an adult population [13,14] favored the Web-based intervention on asthma-related quality of life and on self-reported symptoms and asthma control. Previous work has shown that self-reported asthma control often overestimates the effect of interventions [41]. Our study was the first to examine the impact of a Web-based self-management program on asthma control independent of participant reporting [12]. We used a composite measure of control based on administrative data and an algorithm for overuse of rescue medication and ED use. While none of the previous studies examined the overuse of rescue medication as an outcome, they have evaluated the impact on ED visits. Similar to our study, Rasmussen et al [14] found no difference in ED visits between the intervention and control groups. Unlike our investigation that showed an association between self-efficacy, depression, and beliefs about medications with asthma-related quality of life, none of the previous studies to our knowledge examined these mediating effects as possible mechanisms by which Web-based self-management interventions enhance outcomes [12]. This also reflects the limited consideration of theory defining the core mechanisms by which technology-based interventions impact outcomes and guide the evaluation of self-management interventions [42]. Al-Durra et al (2015) found that only 20% (17/85) studies of Web-based asthma self-management interventions applied at least one model, framework, or construct of a behavioral change theory [43].

Furthermore, a consistent finding across systematic reviews of technology-enabled self-management is that interventions with multiple behavioral change techniques appear, on the whole, to be more effective than those using fewer techniques [12,40]. We also identified this as important during the iterative design process of MAP and in consultation with behavioral change experts on our team and incorporated several techniques within MAP, including self-monitoring, action planning, feedback in relation to symptoms and goals, environmental triggers, motivational messaging, and reward. Future work will focus on tailoring the use of behavioral change techniques and gradually combining techniques in response to individuals’ clinical profile and preferences to help participants identify a sequence of behaviors to target. Tailoring the system to the needs of individuals as they take control of their health may help optimize individuals’ perceived benefit of self-monitoring and staying connected to the care team as needed. In turn, this may increase the ongoing participant use of MAP and an opportunity to observe the long-term effects on asthma control.

### Strengths and Limitations

Linking the mechanisms by which self-management interventions are expected to have an effect is important to guide the selection of functionalities that should be included in technology-based interventions. The features and decision support tools in MAP are based on the principles of self-management skill development [44], self-efficacy and motivational theory [45], and adult learning principles [44], and were integrated into clinical care with ongoing monitoring by a nurse case manager. These theories and the clinical process for asthma management guided the behavioral change techniques within MAP, the algorithm behind the frequency and nature of alerts to patients, and the interaction with the case manager [15]. While previous systems have included Web-based monitoring, most do not provide a comprehensive evaluation of asthma-related behaviors that can then be used to deliver automated alerts and decision support to patients and the care team [12]. In our study, we targeted asthma symptom monitoring and action plan use, but also included physical activity monitoring, which has been shown to be linked to averting exacerbations in the long term [46].

One of the limitations of this trial was that we did not evaluate adherence to asthma treatment. This limited our ability to evaluate the impact of MAP on adherence as an intermediate effect to improve asthma control. Also, the number of participants lost to follow-up and missing data resulted in a relatively small, but sufficient, sample size. Comparison of baseline characteristics and imputation to evaluate the impact of missing data provided insight into the characteristics of individuals who dropped out, and the implications for feasibility and the need to tailor the intervention. Previous reviews of computer-based asthma interventions reported attrition rates of up to 23% [12]. This is in keeping with other systematic reviews of computer-based interventions in other areas [12,40]. Our study had a higher attrition of 37%, which is still lower than the upper range of attrition found in nontechnology-based self-management interventions, which ranges from 0 to 54% [47,48]. Nonetheless, 16 participants were lost to follow-up in the intervention group compared with only 3 patients lost to follow-up in the control group. This finding implied that dropout attrition rate was more than 5 times higher in the intervention group compared with the control group. Eight individuals among those who dropped out reported a lack of time as a reason, indicating that the use of the system needs to be simplified to optimize use as part of daily activities. The remaining 7 individuals, who could not be reached and found the study time-consuming, might not have perceived MAP as beneficial. Initial perceived benefit could be used as a way to screen for potential users of MAP and identify ways of optimizing the fit of MAP to the needs of individuals with asthma. Only one person identified no access to a computer as a reason for dropout, indicating that, for some, lack of access to the Internet remains a potential barrier. Further, participants in this trial might have been those who were more likely to accept the use of technology, which might have limited the generalizability of the results to other patients with chronic conditions.

Another consideration is that care in specialty clinics was more intensive in terms of providing patients with case management and self-management support compared with primary care, which likely contributed to making it more challenging to find an effect. We would expect a larger difference in outcome between the Web-based tool and usual care in a primary care setting. Also, given the relatively small sample for this trial, about half had disease for >10 years and therefore had a greater opportunity to learn and improve asthma self-management behaviors prior to starting the trial. MAP may have greater impact among individuals in relatively earlier phases post-asthma diagnosis when learning self-management skills is new for them. This represents another promising avenue for future research.

Furthermore, the material and recommendations presented to participants in response to monitoring information were static for the entire 6-month period. Adapting the monitoring protocol and material and recommendations based on individuals’ progression and time using the system may enhance usage and impact on outcomes.

Finally, from the time the trial was registered there were changes made to the design prior to the start of the trial, including expanding the inclusion for age from ≤60 to ≤69 and removing the criteria for full healthcare coverage and the need for the primary care physician to be using the asthma decision support.

### Conclusions

There is growing interest in the potential of the Web-based and other digital media as a platform to deliver more tailored self-management support, while maintaining cost-effectiveness, with greater scope for integration into the everyday lives of those with asthma. The MAP intervention had a significant impact on improving asthma-related quality of life and other related outcomes including self-efficacy, depressive symptoms, and beliefs about medication. Furthermore, the effects of MAP were maintained at 6 months and began to decrease once the use of MAP ended between 6 and 9 months, suggesting that ongoing support might result in reduced exacerbations in the long term as opposed to usual care alone. Technology renders self-management support between clinical visits feasible. Future work is needed to identify tailored protocols for monitoring symptoms and health behaviors as individuals learn how to self-manage, and to assess the impact of MAP on asthma control and health-related quality of life beyond 6 months. Based on these results, a larger-cluster randomized trial will be designed with an updated version of MAP that will allow us to evaluate the long-term impact of tailored self-management, adjusting for multiple covariates over time.

## Multimedia Appendix 1

My Asthma Portal (MAP) monitoring business rules.

## Multimedia Appendix 2

Changes in asthma quality of life and asthma control status over time.

## Abbreviations

ACT: Asthma Control Test
ED: emergency department
EQ-VAS: EuroQol visual analogue scale
MAP: My Asthma Portal
MAQLQ: Mini-Asthma Quality of Life Questionnaire
PHQ: Patient Health Questionnaire
