# Supplementary material for: The Effectiveness of Web-Based Asthma Self-Management System, My Asthma Portal (MAP): A Pilot Randomized Controlled Trial
Source: J Med Internet Res. 2016 Dec 1;18(12):e313. doi: 10.2196/jmir.5866 (PMC5159614; doi:10.2196/jmir.5866)
Supplement: Supplementary file 1 [file jmir_v18i12e313_app1.pdf]

Outcome Monitored	Green	Yellow	Red
Symptoms	No symptoms	1 symptom	2 symptoms or emergency visit or both
Overuse of rescue puffer[a]	P<150	250>P≥150	P≥250
Adherence to preventative medication[b]	n/d≥90%	90%>n/d>70%	n/d≤70%
Exercise[c]	number steps ≥ average	number steps > 2/3 of average	number steps < 2/3 of average
Action Plan Understanding%	"Yes" to all questions	N/A	"No" to at least one question

[a](*P* = number of puffs corresponding to all FABA and combivent dispensed over the past 3 months).
[b](quantity dispensed/3 months) [144].
[c] Calculated once exercise monitoring questionnaire is completed 4 or more times (average is the mean value for the first three entries, x = the last steps entry).
%Action Plan questions: Do you understand what to do in your action plan? Are you able to describe the signs or symptoms you have when your asthma is getting worse?
