# Supplementary material for: The Effectiveness of Web-Based Asthma Self-Management System, My Asthma Portal (MAP): A Pilot Randomized Controlled Trial
Source: J Med Internet Res. 2016 Dec 1;18(12):e313. doi: 10.2196/jmir.5866 (PMC5159614; doi:10.2196/jmir.5866)
Supplement: Supplementary file 2 [file jmir_v18i12e313_app2.pdf]

**Evaluation Time**	Poor control status						MAQLQ					
	Intervention		Control		Change between time periods[b]		Intervention		Control		Change between time periods[b]	
	n (%)	SD[a]	n (%)	SD	OR[c]	95% CI	n	Mean (SD)	n	Mean (SD)	MC[c]	95% CI
**Observed**												
**Baseline**	45 (15)		45 (18)		0.74	0.25-2.19	47	4.8 (1.3)	51	4.9 (1.3)	–0.10	–0.58 to 0.39
**3 months**	45 (15)		45 (16)		0.67	0.18-2.52	31	5.6 (1.2)	47	5.0 (1.3)	0.47	–0.05 to 0.99
**6 months**	45 (17)		45 (16)		0.62	0.16-2.33	28	5.6 (1.2)	48	5.2 (1.2)	0.31	–0.21 to 0.84
**9 months**	45 (17)		45 (16)		0.69	0.18-2.73	28	5.5 (1.3)	47	5.1 (1.2)	0.15	–0.38 to 0.67
**Imputed**												
**Baseline**	47 (16)	1.6	51 (23)	2.1	0.67	0.23-1.94	47	4.8 (1.3)	51	4.9 (1.3)	–0.10	–0.62 to 0.42
**3 months**	47 (15)	0.9	51(19)	1.3	0.78	0.26-2.28	47	5.3 (1.4)	51	5.0 (1.2)	0.27	–0.28 to 0.81
**6 months**	47 (18)	1.4	51 (19)	1.9	0.94	0.33-2.71	47	5.3 (1.3)	51	5.1 (1.2)	0.22	–0.34 to 0.78
**9 months**	47 (19)	1.4	51 (18)	2.2	1.09	0.37-3.22	47	5.0 (1.5)	51	5.1 (1.2)	–0.08	–0.63 to 0.46

[a]There is an SD on the proportion of patients with poor control status only for imputed data.

[b]Changes reported between intervention and control groups are for models adjusted for intervention group and time only

[c]OR, odds ratio.

[d]MC, mean change.
